# Ultrasound guided intralesional injections of platelet rich plasma in the treatment of iliac crest pain syndrome and the associated low back pain

**DOI:** 10.3389/fmed.2026.1943518

**Published:** 2026-10-23

**Authors:** Plamen Todorov Todorov, Lily Mekenyan, Leonidas Seventekidis, Anastas Batalov

**Affiliations:** 1 Department of Propedeutics of Internal Diseases and Rheumatology, Medical University of Plovdiv, Plovdiv, Bulgaria; 2 Clinic of Rheumatology, Universitetska mnogoprofilna bolnica za aktivno lecenie Kaspela EOOD, Plovdiv, Bulgaria

**Keywords:** enthesopathy, iliac crest pain syndrome, low back pain, musculoskeletal ultrasound, platelet rich plasma, ultrasound guided

## Abstract

**Introduction:**

Iliac Crest Pain Syndrome (ICPS) was established as a discrete and common cause for Low Back Pain (LBP) of soft tissue origin. Previous studies on ICPS proposed enthesopathy of Erector Spinae (ES) muscle and Thoracolumbar fascia (TLF) at the Posterior Super Iliac Spine (PSIS), as well as pathology of the Long Posterior Sacroiliac Ligament (LPSL) as main pathologic substrates in ICPS. Platelet rich plasma (PRP) is an orthobiologic treatment that was found to be especially efficacious in soft tissue pathology, like enthesopathy and ligamentopathy. In this study we aimed to assess the efficacy of PRP intralesional injections at the ES/TLF/LPSL entheses in patients with ICPS and associated LBP.

**Material:**

The study was partially funded by a postdoctoral research grant of the Medical University of Plovdiv and included 46 ICPS patients. Each patient received two intralesional PRP injections in their ES/TLF/LPSL entheses at the PSIS within a three-week interval. All procedures were performed under direct US guidance. The primary endpoint was pain reduction on VAS (0–100) in six months. Secondary endpoints included assessment of the range of motions in the lumbar spine, disability (RMDS: 1–24) and health status (HAQ: 0–3), as well as patients’ satisfaction (five points Likert scale), all in six months.

**Results:**

Pain in rest decreased from 53 to 16 mm, pain on motion—from 69,5 to 18 mm on VAS in six months. Lumbar range of motion increased with the average of 2,5 cm both in the sagittal and coronal planes. Regarding PROs, RMDS dropped from 15 to 6,5 points, and HAQ from 1.13 to 0.62 in six months.

**Discussion:**

In the present study, PRP was applied directly intralesional under US guidance, following a strict and easily reproducible protocol. The results showed a significant positive effect on all the investigated parameters: pain, spinal mobility and PROs. Thus, PRP US guided injections at the PSIS could represent a valid and efficacious treatment option in patients with ICPS and the associated LBP.

## Introduction

Low back pain (LBP) is among the leading causes for medical consultations worldwide (1), but it remains a diagnostic challenge in the rheumatology practice (2). While there is an abundance of literature on the effect of intervertebral discs and joints on LBP, the role of paraspinal soft tissue structures (ligaments, tendons, entheses, fascia, muscles) should further be studied and considered (3, 4).

In fact, soft tissue pathologies lead to numerous regional pain syndromes and their importance to cause or contribute to LBP might have been somewhat neglected because of the widespread use of conventional imaging methods such as radiography and computerized tomography that show primarily the bony elements of the axial skeleton (5). However, with the introduction of newer imaging modalities, like Magnetic Resonance Imaging (MRI) and Musculoskeletal Ultrasound (MSUS), the paraspinal soft tissues can also be assessed in greater detail, and their pathological transformations can be revealed (6). Regarding MSUS in particular, numerous publications confirm that pathologically transformed entheses, tendons and ligaments display a variety of sonographic markers that allow for reliable diagnosis (7).

In patients with LBP, a regional pain over the posterior superior iliac spine (PSIS) is quite common (8). This complaint was found so frequently that a distinct regional pain syndrome was described in 1991, namely the Iliac Crest Pain Syndrome (ICPS) (9). This syndrome is easily identified clinically, based on the typical location of the pain over the PSIS and its reproduction by firm palpation at this site, or by movements of the lumbar spine (9). In a later study, the reliability of the clinical examination was found to be good, but the etiology of this symptom remained obscure (10).

However, there are other entities that could cause pain in this region, like superior cluneal nerve entrapment, sacroiliac joint pain, facet syndrome. Careful clinical analysis could help in recognizing them. The ICPS could be differentiated from Maigne syndrome (Superior cluneal nerve entrapment) by the lack of sensory component in the patient's complaints, as well as the more inferior-medial site of pain peak on palpation (9, 11). In sacroiliac pain there are also positive manual stress tests, and the pain radiates to the gluteal region and the thigh, while radiation is not typical in ICPS (9, 12). The well-defined, relatively superficial site of maximum pain and the lack of radiation and sensory symptoms could be considered as the main discriminators from lumbar facet syndrome (9, 13).

Anatomical studies show that the site of tenderness in ICPS coincides with several muscular and ligamentous attachment sites in this area (3). These include the entheses of the Erector Spinae (ES) muscle and parts of the Erector Spinae Aponeurosis (ESA) and thoracolumbar fascia (TLF) superior-medially, the Gluteus Maximus (GM) inferior-laterally and the Long Posterior Sacroiliac Ligament (LPSL) inferiorly (3).

The TLF is a complex of several layers that separates the paraspinal muscles from the muscles of the posterior abdominal wall, quadratus lumborum and psoas major (14). It forms a compartment around the paraspinal muscles, attaching to the spinous process posteromedially and to the transverse process anterolaterally. Laterally the TLF blends with the aponeuroses of the transversus abdominis muscle and this junction is called lateral raphe. Beginning at approximately L5 and below, the TLF fuses tightly with the underlaying aponeurosis of the ES, making one very thick aponeurotic structure, which attaches laterally to the iliac crest at PSIS (14).

The ES, formed by iliocostalis and longissimus, is the biggest among the back muscles and it consists of a thoracic and a lumbar part (3, 15). In the lower lumbar region, these muscles are completely covered by the dense erector spinae aponeurosis (ESA), which constitutes the conjoint tendon of ES thoracic part (14, 15). The detailed anatomical study of the caudal ES attachments performed by Macintosh and Bogduk showed that the lateral part of ES aponeurosis attaches to the PSIS and the most medial iliac crest, while the medial part continues caudally to attach to the sacrum and the sacrotuberous ligament. Some of the fibers of the deep (or lumbar) part of the ES muscle attach to the inner surface of ESA. However, most of them attach directly to a relatively small area on the medio-superior part of the PSIS, just beneath the ESA attachment site, either as tendinous or direct muscular entheses (15).

The LPSL is the most superficial and thick ligament that supports the sacroiliac joint (SIJ) (3, 16). It spans between the inferior pole of the PSIS and the lateral sacral crest and transverse tubercula of the third and fourth sacral segments (16). Besides stabilizing the SIJ, resisting the counternutation of the sacrum during walking and standing, it also serves as an important linking point between the myofascial systems of the lower back and the gluteal regions, as both the erector spinae aponeurosis (ESA) and the gluteus maximus aponeurosis (GMA) have attachments on the LPSIL (16) (Figure 1, Supplementary Figures 10A,B).

**Figure 1:**
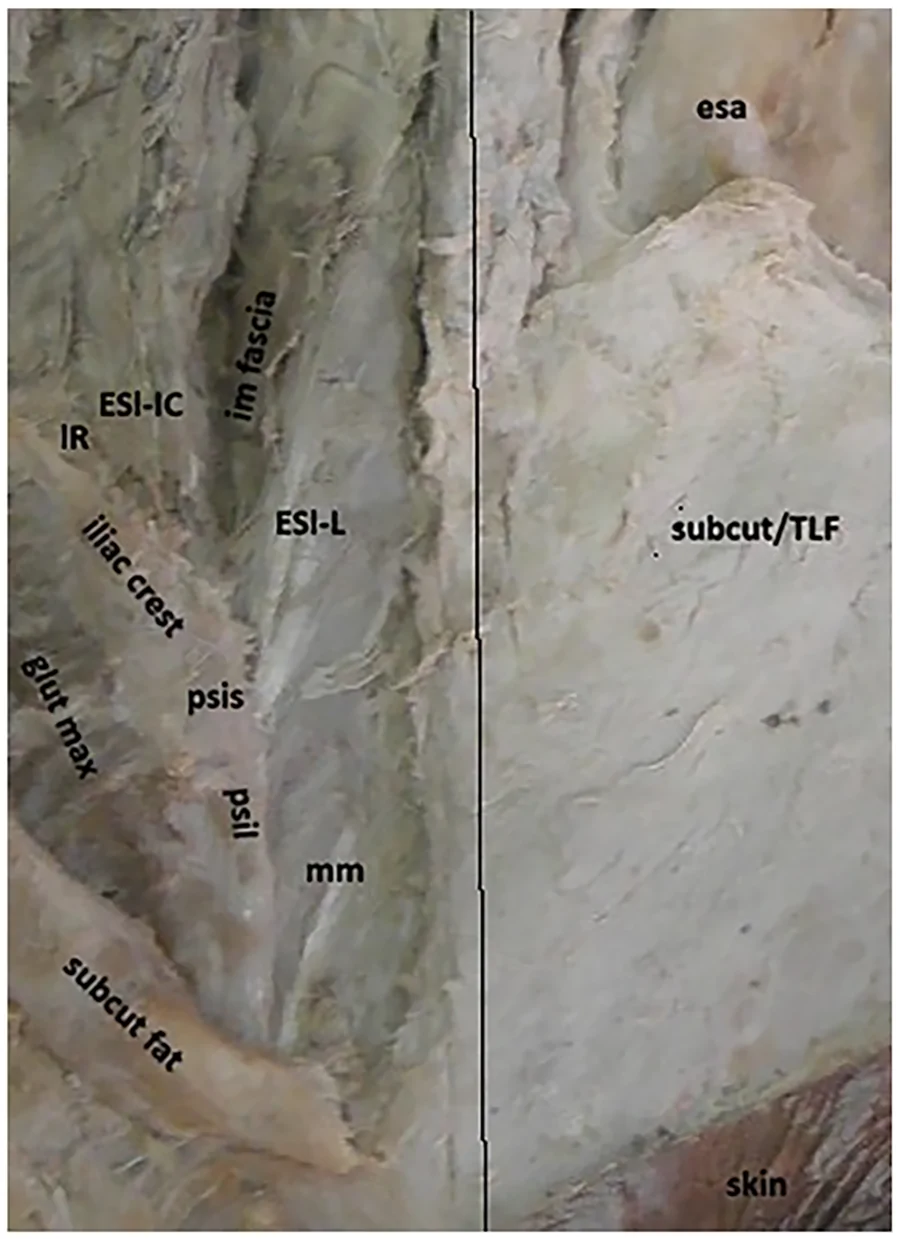
Cadaver dissection showing the anatomy of the entheses around the PSIS. On the left side the TLF and the ESA are both removed, revealing the iliac crest and the PSIS, as well as ES lumborum muscles—(Iliocostalis lumborum laterally and Longissimus medially) attaching to them from above and the PSIL attaching to the inferior pole of the PSIS. Note that the Iliocostalis is pushed laterally to exhibit also the intramuscular fascia. The lateral raphe is seen just lateral of Iliocostalis lumborum and the Gluteus maximus is seen caudal of the iliac crest, while the multifidus muscle emerging under the most medial part of longissimus to cover the sacrum. On the right side of the image the TLF and ESA are preserved and cover the structures displayed on the left side of the cadaver. Black vertical line represents the midline. PSIS, posterior superior iliac spine; LPSL, long posterior sacroiliac ligament; ESL-IC, erector spinae lumborum-Iliocostalis; ESL-L, erector spinae lumborum-longissimus; Glut max, gluteus maximus; MM, multifidus Muscle; ESA, erector spinae aponeurosis; TLF, thoracolumbar fascia.

Interesting data about the nature and changes of the soft tissue structures around the PSIS have emerged from a surgical study that explored a new technique for SIJ debridement and fusion (17). Sections of the removed tissues from the PSIS area were histologically explored as a part of this study and revealed a non-inflammatory degenerative condition of “the extra-articular tendinous attachments”. The authors pointed out that: “the observed histological pattern is the same as seen in lateral epicondylitis” and concluded that these changes could be the source of the complaints in at least some patients with SIJ regional pain (17).

Previously, in anatomical and ultrasound studies performed by our group, we showed that the attachments of ES muscle, the ESA and the TLF could be visualized and separately assessed in detail by the means of high resolution MSUS (18, 19). The LPSL, being relatively superficial, was also shown to be easily assessable by MSUS both by our group and confirmed by other researchers as well (20, 21). In addition, several studies confirmed the presence of US-identifiable enthesopathies of the ES and TLF at the PSIS (22–24). As there is a good acoustic window to these structures, they could be targeted precisely by MSUS guided interventions (22, 23).

Platelet rich plasma (PRP) is an orthobiologic treatment that was found to be particularly efficient in soft tissue degenerative pathology like tendopathies, enthesopathies and ligamentopathies (25). Histology proved that PRP treated structures show better collagen organization and increased metabolic activity (26). In addition, many studies reported promising results and clinical efficacy of PRP infiltration in several soft tissue pathological conditions, like rotator cuff injuries (27), lateral and medial epicondylalgia (28), patellar and Achilles tendopathies (29), ulnar collateral ligament of the elbow (30), cruciate ligaments of the knee (31), to name just a few. In general, as PRP administration shows low risk and favorable results, it represents a viable conservative treatment of soft tissue rheumatological conditions (32). The data on the efficacy of PRP treatment in LBP is mostly from studies on its intradiscal application, while there is relatively few evidence on its possible therapeutic effect in soft tissue paravertebral structures pathology (33).

## Objectives

The aim of the present study was to assess the efficacy of ultrasound guided intralesional administration of PRP in LBP patients with clinical and ultrasound signs of ICPS.

## Material and methods

The study was performed in a tertiary university hospital rheumatological department and was partially sponsored by a postdoctoral research grant (19/2019) of the Medical University of Plovdiv.

*Inclusion criteria*: adults above 18 years of age, LBP >3 months with a punctum maximum of the pain at the PSIS, conformation of the presence of ICPS both clinically (i.e., reproduction of the typical patients' pain upon firm palpation over the area of the PSIS) and sonographically (sonopathology of the ES, ESA and/or TLF entheses at the PSIS, and/or the LPSL).

*Exclusion criteria*: any inflammatory, neoplastic or traumatic conditions of the spine and pelvis, presence of osteoporosis with vertebral or hip fractures, L5/S1 facet joint and disc diseases, hematological condition, presence of sciatica or neuropathic pain, usage of anticoagulants or current infectious disease, administration of local cortisone injections in the lumbo-pelvic area in the previous three months.

In addition, patients who met these criteria and were deemed eligible for inclusion in the study were sent for a laboratory check of their peripheral blood (platelet count and Erythrocytes Sedimentation Rate (ESR). To proceed and be finally included in the study, the patients should have more than 200 × 10^9^ platelets in the peripheral blood and an ESR not higher than 25 mm/h. This last inclusion criterion was based on the foundational definition of PRP as “a concentration of 1 million platelets/µL in 5 mL of plasma” (34) and further publications (35, 36) that also set this threshold for the number of platelets in the final product to be defined as PRP. As the manufacturer specifications of the PRP preparation kits we used for the study (SW-PRP, Seawon Meditech Co, South Korea) certified a 5.5 time increase in the platelets count after the centrifugation process, we used the cut off of 200 × 10^9^ platelets to ensure an adequate baseline number of platelets before the concentration process. Regarding the ESR value <25 mm/h, it did not only help to rule out possible inflammatory and/or infection conditions (the presence of such conditions was excluded based on the check of the patients' medical documentation, history taking and the clinical examination before inclusion), but also helped to set a more homogeneous study population, as it is known that factors influencing ESR can affect PRP sedimentation dynamics even in the absence of inflammation (37).

### Clinical assessment

After signing an informed consent form, each patient's documentation was carefully reviewed to confirm the absence of any of the exclusion criteria. Then patients underwent standard musculoskeletal examination of the lumbar spine, pelvis and hip joints in erect, supine and prone position. The spinal range of motion (ROM) was recorded for both the forward (modified Schober test) and lateral (fingers to ipsilateral fibula head distance) lumbar flexion. Intensity of pain either at rest and in motion was recorded separately on a Visual Analogue Scale (VAS).

### MSUS assessment

After the clinical examination, a standardized MSUS assessment of each patient was performed. It followed the protocol we previously elaborated and validated (18, 20). Esaote My Lab ×8 with a multifrequency linear transducer (5–15 MHz) was used for all assessments and procedures. The patients were placed in a comfortable prone position and longitudinal, oblique and transverse scans at the level of PSIS were performed (Figure 2), assessing the ES, ESA and TLF entheses at the superior pole of the PSIS (Figures 3A, 4) and the LPSL at the inferior (Figure 5). Features indicative of a pathology of these structures include thickening, hypoechogenicity, altered fibrillar pattern, internal anechoic areas, hyperechoic foci, as well as irregularities of the subentheseal bony contour of the ES, ESA and TLF entheses, i.e., enthesopathy (Figures 6, 7) and ligamentopathy of the LPSL (Figure 8) according to the official guidelines for the MSUS assessment of extraarticular soft tissues published by EFSUMB (7). Representative images of each scanning were stored on an external device.

**Figure 2:**
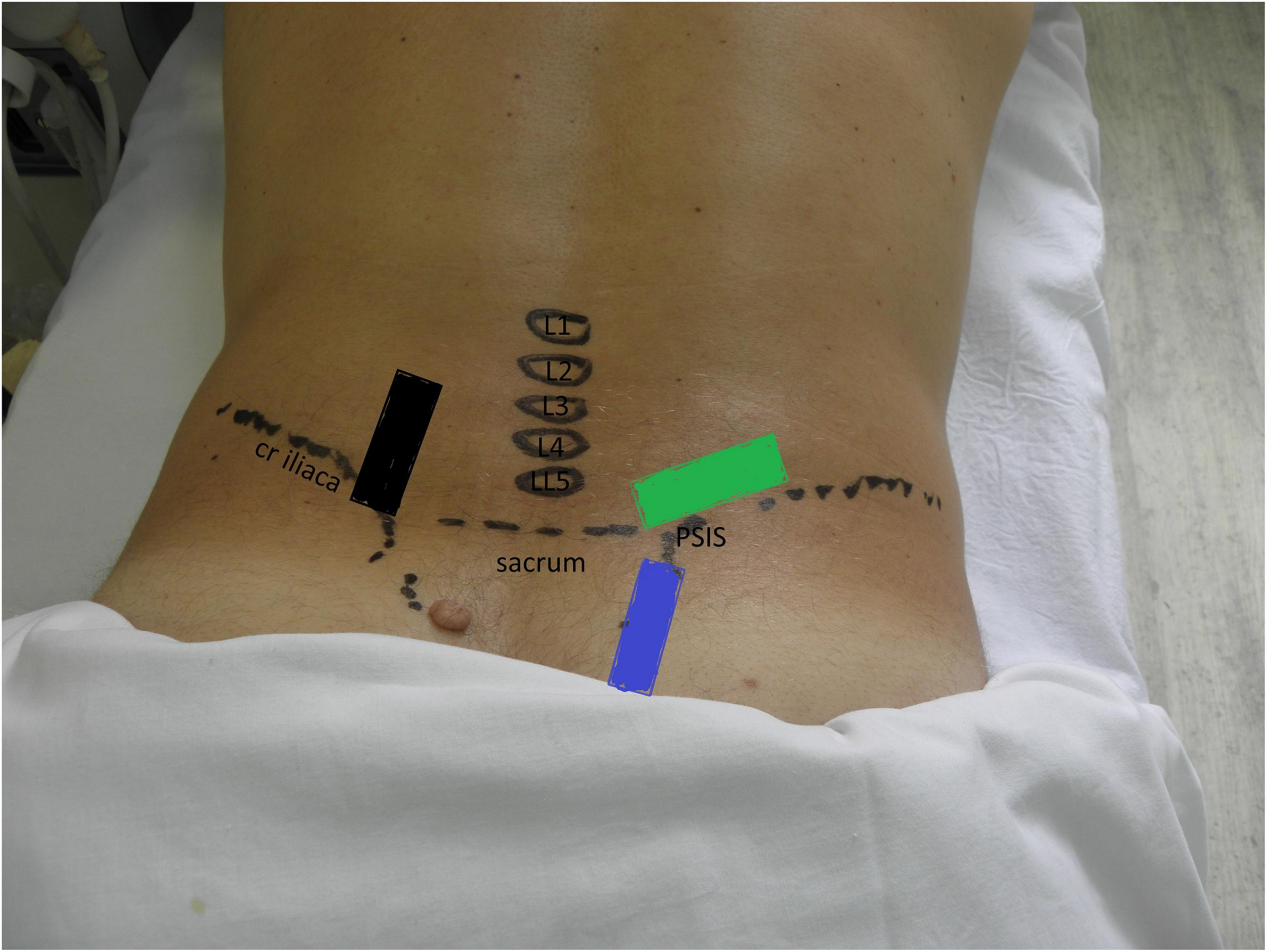
Scanning positions for the US assessment of: erector spinae/thoracolumbar fascia in the longitudinal (black) and the transverse (green) planes and the long posterior sacroiliac ligament in the longitudinal plane (blue).

**Figure 3:**
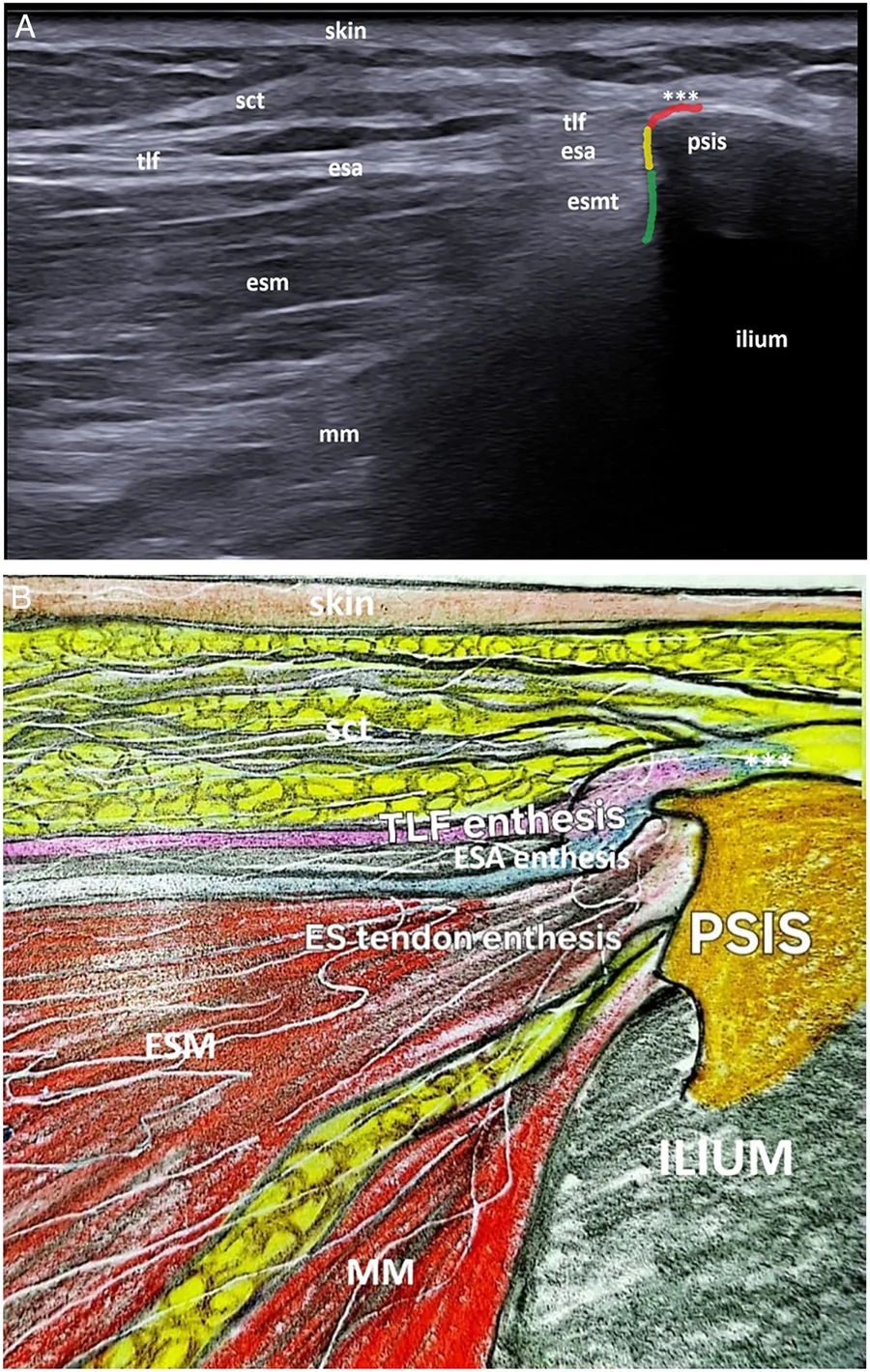
**(A)** ultrasound image of a normal erector spinae/thoracolumbar fascia enthesis at the posterior superior iliac spine in the longitudinal plane: the TLF part of the entheses is most cranially at the PSIS, indicated by a red line; the ESA enthesis is marked in yellow and the lumbar ESM tendon enthesis is most caudally and indicated by a green line. sct, subcutaneous tissue; esm, erector spinae muscle; mm, multifidus muscle; tlf, thoracolumbar fascia; esa, erector spinae aponeurosis; esmt, erector spinae muscle tendon; psis, posterior superior iliac spine; *** indicate the part of the TLF that continues caudally not attaching to the PSIS. **(B)** Representative drawing of the Erector Spinae/Thoracolumbar fascia enthesis at the Posterior Superior Iliac Spine in the longitudinal plane.

**Figure 4:**
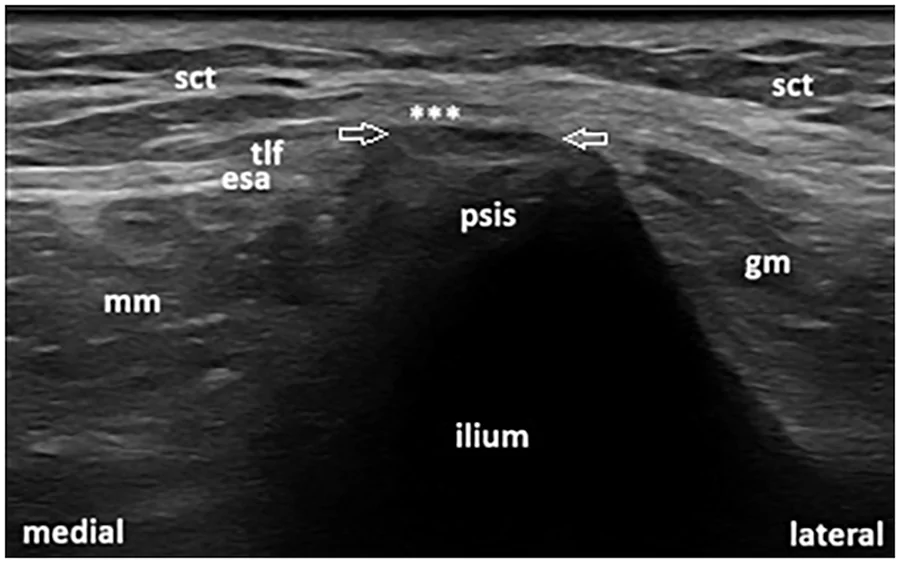
Ultrasound image of a normal erector spinae/thoracolumbar fascia enthesis at the posterior superior iliac spine in the transverse plane—between the arrows. sct, subcutaneous tissue; mm, multifidus muscle; gm, gluteus maximus muscle; tlf, thoracolumbar fascia; esa, erector spinae aponeurosis; psis, posterior superior iliac spine, *** indicate the part of the TLF that continues caudally not attaching to the PSIS.

**Figure 5:**
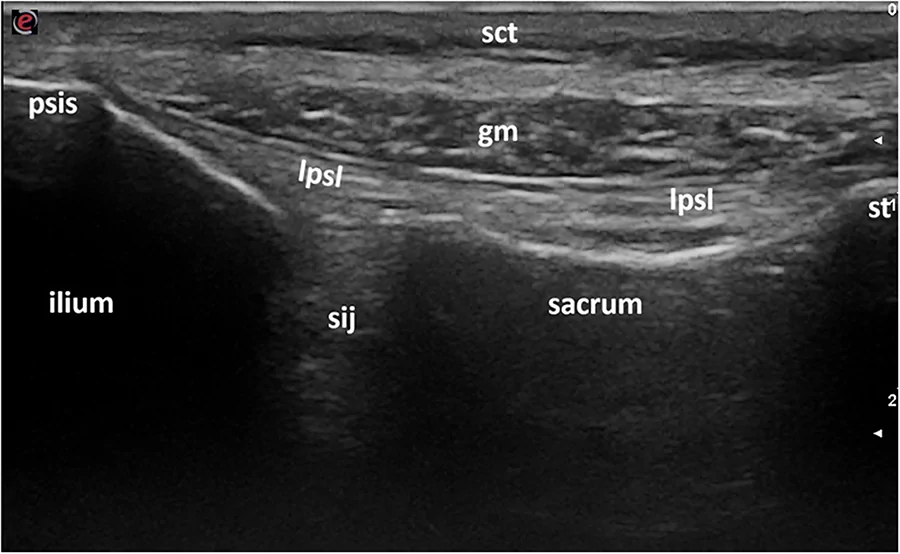
Ultrasound image of the long posterior sacroiliac ligament in the longitudinal plane. sct, subcutaneous tissue; lpsl, long posterior sacroiliac ligament; gm, gluteus maximus muscle; psis, posterior superior iliac spine; sij, sacroiliac joint; st, sacral tuberculum.

**Figure 6:**
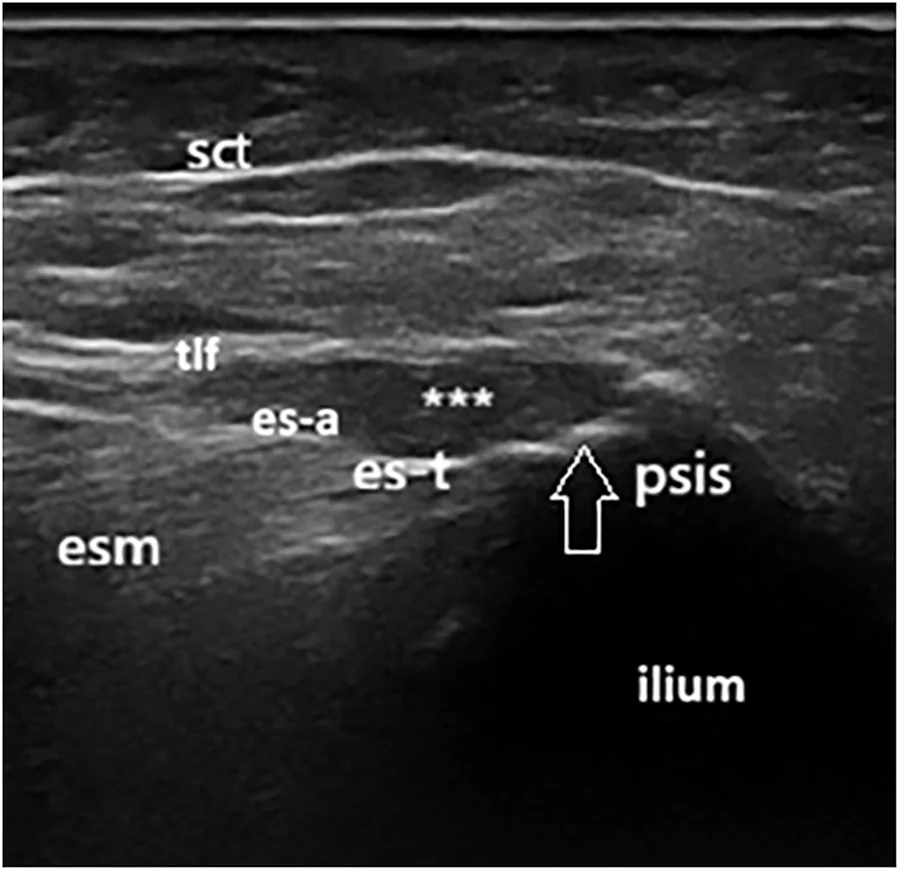
Ultrasound image of an erector spinae/thoracolumbar fascia enthesopathy at the posterior superior iliac spine in the longitudinal plane. sct, subcutaneous tissue; esm, erector spinae muscle; tlf, thoracolumbar fascia; es-a, erector spinae aponeurosis; es-t, erector spinae muscle tendon; psis, posterior superior iliac spine; *** hypoechoic thickening/swelling of the ESA and EST entheses; arrow indicates bony irregularities at the PSIS.

**Figure 7:**
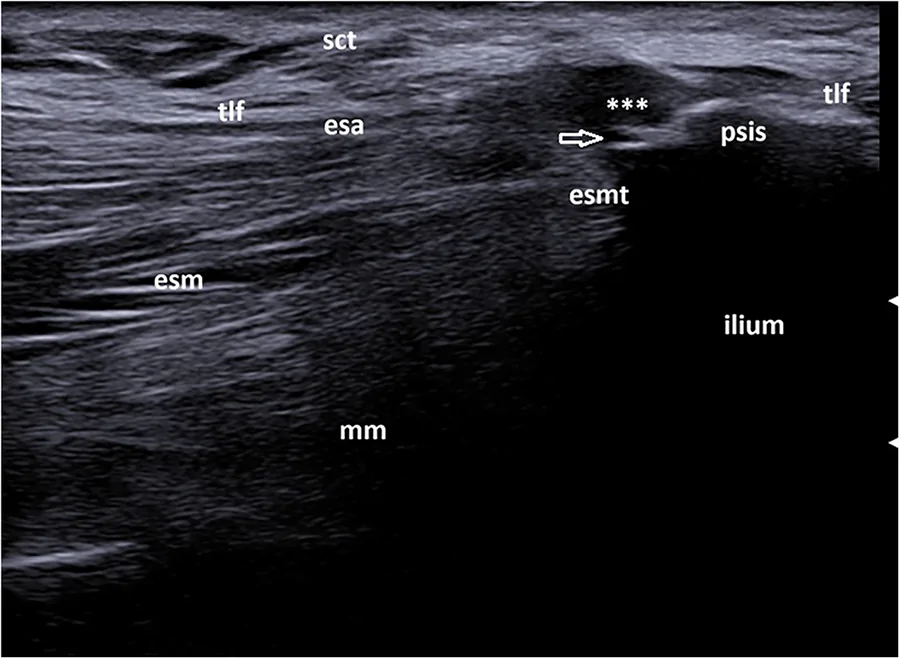
Ultrasound image of an erector spinae/thoracolumbar fascia enthesopathy at the posterior superior iliac spine in the longitudinal plane. sct, subcutaneous tissue; esm, erector spinae muscle; mm, multifidus muscle, tlf, thoracolumbar fascia; esa, erector spinae aponeurosis; esmt, erector spinae muscle tendon; psis, posterior superior iliac spine; *** hypoechoic thickening/swelling of the ES/TLF entheses; arrow indicates enthesophytes at the PSIS.

**Figure 8:**
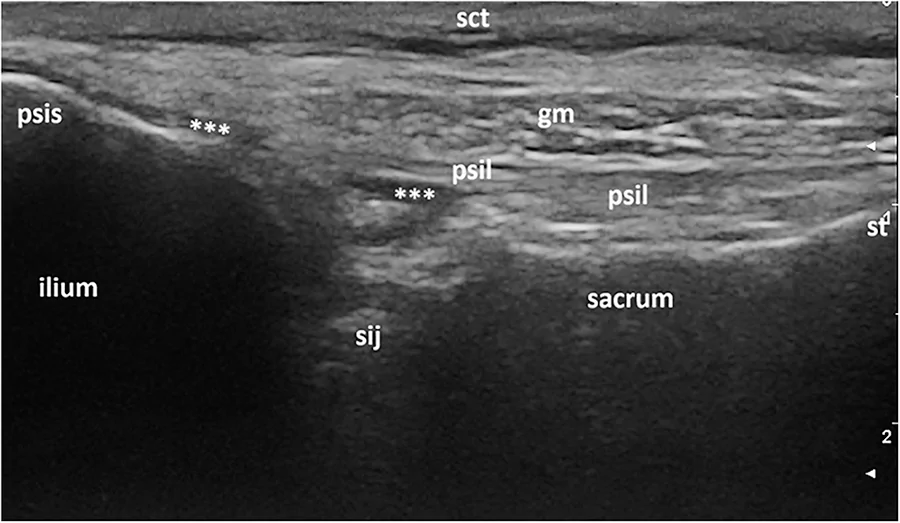
Ultrasound image of a pathological long posterior sacroiliac ligament in the longitudinal plane. lpsl, long posterior sacroiliac ligament; gm, gluteus maximus muscle; psis, posterior superior iliac spine, sij, sacroliac joint, *** hypoechoic zone and altered fibrillar structure at the cranial part and the enthesis of the LPSL at the inferior PSIS.

A total of 55 ICPS patients were identified, selected, assessed clinically and sonographically to confirm the diagnosis and signed an informed consent form. After that a laboratory check of the peripheral blood was performed. It showed that 9 of these patients could not proceed in the study due to either lower than required platelets count or higher than required ESR and were duly excluded.

Thus 46 patients were finally included in the study and received the interventions. The mean age of the study population was 59.67 ± 12.09 years and female patients (84.80%) predominated in the group. Each included patient received two MSUS guided PRP intralesional injections at the PSIS, targeting the ES/TLF entheses and the LPSL within a three-week interval. The primary endpoint was pain intensity, at rest and in motion, on the Visual Analogue Scale (VAS: 0–100) in six months. Secondary endpoints were range of motion in the lumbar spine in sixth months; disability self-assessment (Rolland Moris Disability Score (RMDS: 1–24) and health status (Health Assessment Questionary (HAQ: 0–3) in sixth months; VAS in three weeks and patients' satisfaction from the treatment (five points Likert scale: 1–5) in three weeks and in six months.

At the time of the first procedure, all patients filled in the Patients Reported Outcomes (PROs) forms—VAS (separately for pain at rest and in motion), RMDS and HAQ.

### PRP interventions

Two procedures, three weeks apart, were performed. Each time the same procedure was followed. After cleaning the skin, a 20 mL of venous blood was drawn in a 30 mL sterile syringe (dicoNex), prefilled with 3 mL ACD-A anticoagulant and connected to a special, individual PRP preparation kit (SW-PRP, Seawon Meditech Co, South Korea), with an FDA approval since 2013. Then the blood was mixed with the ACD-A, the three-way valve was turned in the direction of the device, and the solution was slowly injected into it. As per manufacturer protocol, the blood underwent a double spin closed process (7 and 4 min respectively) and the produced PRP (about 2 mL) was drawn from the device's PRP extraction chamber into a sterile 5 mL syringe (dicoNex). Manufacturer specifications showed a 5.5 time increase in the platelets count after the centrifugation process, producing a leukocyte-rich PRP, that did not require additional activation. Thus, based on the PAW classification of PRP, the final product designation would be P3B (35). Taking into account the mean number of platelets in the study population (Table 1), this gives an average number of 1,262,800,000 platelets/mL in the final product, or a total count of 2,525,600,000 (2.53 billion) platelets in the injection syringe per procedure. Thus, the number of platelets delivered for the whole therapy (two procedures × 2 mL PRP) was approximal 5,051,200,000 (5.05 billion).

**Table 1:** Basic patient data.

Value	Statistics
Age	
Mean (±SD)	59.67 ± 12.09
Min–Max	31–76
Gender	
Female	39 (84.80%)
Male	7 (15.20%)
BMI	
Mean (±SD)	28.25 ± 5.20
Min–Max	19.20–38.00
Body side	
Left	26 (56.50%)
Right	20 (43.50%)
Pain duration (months)	
Mean (±SD)	16.15 ± 11.12
Median (IQR)	12 (18)
Min–Max	6–48
Number of platelets (x10^9^)	
Mean (±SD)	229.59 ± 40.65
Min-Max	209.32–300.11
ESR (mm/h)	
Mean (±SD)	9.24 ± 2.87
Min–Max	5.00–20.00

Then, the obtained 2 mL of PRP was injected under direct US guidance in a prone patient position using a free hand in plane approach and a sterile coupling gel. The needle was advanced to the region of PSIS from medially to laterally and aimed directly at the pathological structures (i.e., intralesional application)—the ES/TLF enthesopathy, or the PSIL ligamentopathy. Firstly, the ES/TLF enthesis was targeted and injected as it is located more superficially and then the needle was advanced deeper and angled more caudally towards the ligament. The whole amount of PRP was injected under direct and constant visual control (Figures 9A–C, Supplementary Videos 1, 2, 3, 4). As the ES/TLF enthesis is a larger anatomical structure than the PSIL (3), approximately 2/3 of the volume was injected there. All interventions were performed by the same physician (PT) which guaranteed the consistency of the procedures throughout the study. After the procedure, the patients stayed in the clinic for approximately 30 min to be monitored for any potential side effects.

**Figure 9:**
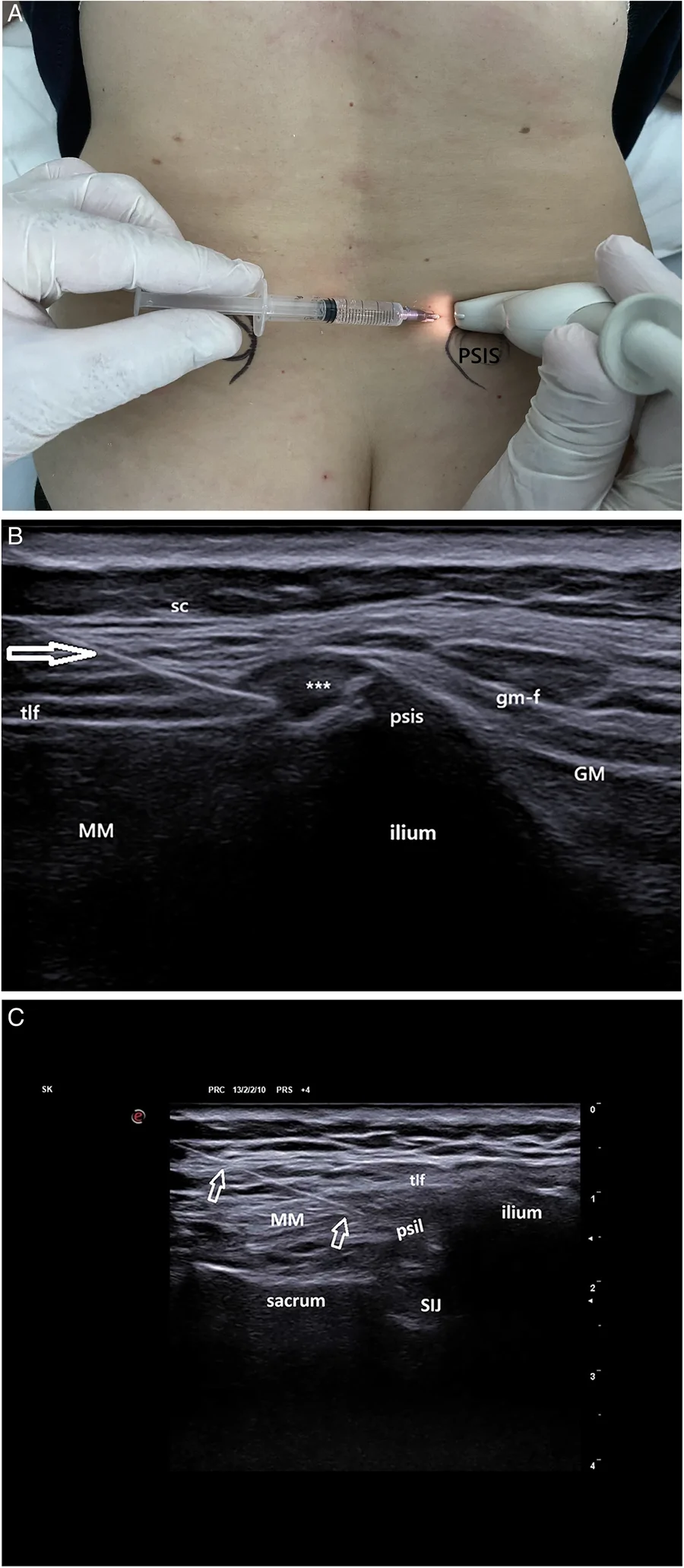
**(A)** US guided PRP injection at the PSIS, in plane, medial to lateral approach. **(B)** Ultrasound image of a PRP intralesional injection in ES/TLF enthesopathy. sct, subcutaneous tissue; mm, multifidus muscle, tlf, thoracolumbar fascia; esa, erector spinae aponeurosis; est, erector spinae muscle tendon; psis, posterior superior iliac spine; gm, gluteus maximus muscle; *** hypoechoic thickening/swelling of the ES/TLF entheses; arrow points to the needle. **(C)** Ultrasound image of a PRP intralesional injection in the Long Posterior Sacroiliac Ligament. mm, multifidus muscle; tlf, thoracolumbar fascia; sij, sacroiliac joint; psil, posterior sacroiliac ligament; arrow points to the needle.

Upon leaving the clinic, patients were instructed to avoid Non-Steroid Anti-Inflammatory Drugs (NSAIDS) for 5 days, simple analgesics (Metamizole) were allowed for pain relief. No specific exercise program or activity restrictions were advised, instead patients proceeded with their normal everyday living and work.

After three weeks, each patient returned to the clinic for the second procedure. Any side effects from the first one were recorded. Before the second injection, patients filled in the VAS form for the current pain at rest and in motion, as well as a five-point Likert scale to rate their satisfaction with the first procedure. After that, the second PRP intralesional injection was performed following the same protocol as for the first one.

## Statistical analysis

Statistical analyses were performed using IBM SPSS Statistics (Version 27, IBM Corp., Armonk, NY, USA). Continuous data were tested for normality using the Shapiro–Wilk test (*p* > 0.05) and reported as mean and standard deviation (SD) for normal distributions or median and interquartile range (IQR) for non-normal distributions. Pain severity across three time points (baseline, Week 3, and 6 months) was analyzed using the Friedman test with *post hoc* pairwise comparisons. Comparisons between two time points were made using the Wilcoxon signed-rank test. Effect size for the Wilcoxon signed-rank test was calculated by dividing the absolute z-statistic by the square root of the total number of paired observations (*n* = 46). Values were evaluated using Cohen's (1988) thresholds for small (*r* = 0.10), medium (*r* = 0.30), and large (*r* = 0.50) effects. Statistical significance was set at alpha = 0.05.

## Results

The study included 46 patients, among whom women prevailed (84.80%). All these patients completed the study and there were not any losses or withdrawals. The average age of the patients was 59.67 ± 12.09 years, with an age range from 31 to 76 years Table 1.

### Change in the severity of pain on VAS between baseline, the third week and in six months

The level of pain on VAS was assessed on baseline, week 3 and after 6 months. For all measurements, the data distribution showed no normal distribution (*p* < 0.01 or *p* < 0.001 at individual time points). The results of the statistical comparison between time points are summarized in Table 2.

**Table 2:** Dynamics in pain intensity between baseline and week 24.

Variables	Baseline	Week 3	Week 24	*p*-value		Effect size *r*
				Friedman test	Wilcoxon test	
VAS (rest)						
Median (IQR)	53 (27)	28 (21)	16 (28)	<0.001	BL → W3: <0.001 BL → W24: <0.001	0.576 0.882
Min–Max	8–100	0–100	0–84		W3 → W24:0.039	0.305
VAS (motion)						
Median (IQR)	69.50 (28)	33 (46)	18 (43)	<0.001	BL → W3: <0.001 BL → W24: <0.001	0.544 0.865
Min–Max	26–100	10–100	7–90		W3 → W24 :0.029	0.227

BL, baseline; W, week; VAS scale: 0 to 100; Effect size (*r*) according to Cohen's reference values: 0.10—small effect, 0.30—medium effect, 0.50—large effect.

For VAS at rest, a significant reduction in pain was found (a drop from 53 to 28 mm) between baseline and week 3—a difference of 25 points, representing a 47.16% reduction in pain level, with a large effect (*r* = 0.576, *p* < 0.001). A further reduction of additional 12 points was recorded between week 3 and week 24 with a medium effect (*r* = 0.305, *p* = 0.039). In total, the change in VAS between baseline and the final visit was 37 points, representing a reduction in pain intensity at rest with 69.80% with a very large effect (*r* = 0.882, *p* < 0.001.

Pain intensity with spinal movements showed a similar pattern, with a significant decrease of 36.5 points (52.50%) between baseline and week 3, showing a large effect (*r* = 0.544, *p* < 0.001). At week 24, pain level decreased by 51.50 points (74.10%), with a very large effect (*r* = 0.865, *p* < 0.001). The change between week 3 and week 24 was also significant, but with a small- to-medium effect (*r* = 0.227, *p* = 0.029) (Table 2).

### Change in the ROM of the lumbar spine assessed by the modified Schober test for the forward flexion and the third finger to ipsilateral fibulae head distance for the lateral flexion

The Schober test had a median of 17.50 cm at the baseline, which is below the minimum criterion value of 20 cm. After 24 weeks a significant improvement in forward flexion was recorded, as the median equals the criterion value of 20 cm, representing an increase of 14.29%, showing a medium-to-large effect (*r* = 0.435, *p* < 0.001.

The lateral flexion, measured by the distance between the tip of the third finger and the head of the ipsilateral fibula, has a median of 7.50 cm at the baseline, which decreases to 5.0 cm after 24 weeks, with a difference significance with a large effect (*r* = 0.532, *p* = 0.001 (Table 3).

**Table 3:** Dynamics in lumbar mobility between baseline and week 24.

Variables	Baseline	Week 24	Wilcoxon *p*-value	Effect size *r*
Schober (cm)				
Median (IQR)	17.50 (2)	20.00 (1)	<0.001	0.435
Min–Max	10–21	16–21		
Lateral flexion (cm)				
Median (IQR)	7.50 (7)	5.0 (9)	0.001	0.532
Min–Max	0–22	0–16		

Effect size (*r*) according to Cohen's reference values: 0.10—small effect, 0.30—medium effect, 0.50—large effect.

### Change in the evaluated PROs (RMDQ and HAQ)

The disability due to LBP, assessed by the RMDQ, showed a significant improvement between baseline and week 24. The median value dropped from 15 to 6.50 points through this period, representing an improvement of 56.67%, with a medium-to-large effect (*r* = 0.413, *p* = 0.005). The health status of patients, assessed by HAQ, also showed a significant improvement. The total score decreased from 1.13 to 0.62, with a difference of 45.13%, with a large effect (*r* = 0.566, *p* < 0.001 (Table 4).

**Table 4:** Dynamics of the assessed PROs between the baseline and week 24.

Variables	Baseline	Week 24	Wilcoxon *p*-value	Effect size *r*
RMDQ (1–24)				
Median (IQR)	15 (10)	6.50 (13)	0.005	0.413
Min–Max	2–19	1–16		
HAQ (0–3)				
Median (IQR)	1.13	0.62	<0.001	0.566
Min–Max	0–2	0–2		

Effect size (*r*) according to Cohen's reference values: 0.10—small effect, 0.30—medium effect, 0.50—large effect.

### Assessment of patients' satisfaction from the treatment at weeks 3 and 24

Patients' satisfaction with the implemented treatment was assessed by a five-point Likert scale where 5 corresponded to complete satisfaction and 1 to complete dissatisfaction. At week 3, before the second intervention, the median value of the Likert scale was 4. At the end of the study period (week 24), the median retained the same value. No significant difference was found between the two time points (*p* = 0.226), indicating that the level of satisfaction observed at week 3 persisted over a longer period (Table 5).

**Table 5:** Level of patients' satisfaction from the PRP therapy.

Satisfaction	Week 3	Week 24	Wilcoxon *p*-value	Effect size r
Median (IQR)	4 (2)	4 (1)	0.226	0.186
Min–Max	2–5	3–5		

Scale: 1 = dissatisfied; 5 = completely satisfied; Effect size (*r*) according to Cohen's reference values: 0.10—small effect, 0.30—medium effect, 0.50—large effect.

## Discussion

ICPS is a regional pain syndrome, which was found to be particularly frequent in LBP (9). The main anatomical substrate behind this syndrome were proposed to be the ES and the ESA entheses at the iliac crest and PSIS (18, 19, 22), as well as the TLF (23), ILL (24) or PSIL (20, 38). All these soft tissue structures could undergo pathological transformation and cause pain in the region of the PSIS—the hallmark feature of the ICPS. Furthermore, the pathological transformation of the soft tissues around the PSIS was generally confirmed in a surgical and histological study (17).

Treatment options in ICPS consist of rest, analgesics, NSAIDS, acupuncture, physiotherapy, with corticosteroid/lidocaine local injections in more severe cases. We were unable to find any studies on the efficacy of physiotherapy or targeted drug regimens on ICPS, probably because largely this condition is included in the umbrella term “nonspecific low back pain” and treated accordingly by the practicing clinicians (4).

Regarding interventions in ICPS, besides case studies (22), a recently published retrospective study showed efficacy of local cortisone injections particularly in the area of TLF entheses (23). There is also a published short term randomized doble blind study on local injections with lidocaine vs. saline in the area of PSIS, showing superiority of the lidocaine injections (39). This indirectly confirms that the pain in ICPS comes from a local source at the PSIS, rather than being referred from other spinal structures.

PRP is widely used in the clinical practice for regeneration and restoration of tissues (40). Recently, especially in the field of sports medicine and orthopedics, the regenerating potential of PRP has been demonstrated when restoring damaged tissues, including tendons, ligaments, and cartilage—all these structures have inherently low healing potential due to their relative avascularity (41). Consequently, a potentially good effect of the administration of PRP in the tendinous and ligamentous tissues around the medial iliac crest and PSIS in patients with ICPS could be expected (33).

In our study, PRP applied directly intralesional under real time US guidance at the ES, ESA and TLF entheses and the LPSL, showed a significant positive effect on all of the investigated parameters: pain, spinal mobility and PROs. The effect on pain was noted even after the first procedure and augmented further after the second. This effect stayed till the end of the study period, i.e., lasted at least 24 weeks.

At the same time spinal mobility increased as assessed by both forward and lateral flexion. Worth noting is that the forward flexion (modified Schober test) reached normal values at the end of the study, while the increase in lateral flexion, though recorded, was not so prominent. This fact might be due to different structures that are involved or stretched during forward and lateral flexion of the lumbar spine. While the TLF, ESA, ES muscles and tendons, and the PSIL are mostly stretched in forward flexion, the Quadrates lumborum muscles, the facet joints capsule and the ILL are predominantly stretched during lateral flexion. Thus, the most pronounced effect of the procedure on forward flexion might confirm the direct effect of PRP in the injected tissues, as the site of injections involved the ES/TLF entheses, cranial, and the PSIL caudal of PSIS.

As self-assessment tools we used RMDQ and HAQ. RMDQ is designed to assess the disability in daily living caused specifically by LBP. Its score is from zero to twenty-four points, with more points representing greater disability. RMDQ was validated in many studies analyzing the effect of a given therapy for LBP (42).

HAQ is a tool used to assess health-related quality of life, particularly in rheumatology. It helps identify difficulties, track progress, and plan better care by evaluating various dimensions such as disability, pain, and medication effects (44). The patient responds on a scale of 0 (no disability) to 3 (completely disabled). A score between 0 and 1 indicates mild to moderate disability, 1–2 indicates moderate to severe disability, and 2–3 indicates severe to very severe disability. In addition, the HAQ was proved to be sensitive to change, which makes it suitable to evaluate therapeutic effects (43).

In our patients these PROs were analyzed at two time points: at baseline and at the end of the study (week 24). Both instruments showed significant improvement: RMDQ by 43%, HAQ by 55% with the HAQ score actually representing a change in the category of the patient's disability, switching it from moderate (score 1.13) to mild (score 0.62).

Finally, all patients were asked about their satisfaction with the PRP treatment at two time points—at week 3 (i.e., when only one procedure was done) and at week 24. We used 5-points Likert scale and at both time points the value was FOUR, showing that patients were predominantly satisfied with the effect of PRP intralesional therapy.

There are several other studies evaluating the effect of the administration of PRP in the paraspinal soft tissue structures in LBP. The available initial results concerning the use of PRP in these structures are encouraging as for the other pain generators in the lumbo-sacral spine. Anyway, the difficulty in analyzing the data is even greater here, given the heterogenicity of the treated structures, and the lack of image guidance for interventions, i.e., lack of proof for the adequacy of administration of PRP exactly in the target structure.

Darrow et al. (44) reported results of a prospective study involving 67 patients with chronic, “non-specific” lumbar pain persistent after standard treatment. These patients received one or two (at an interval of 24 days on average) injections with PRP around the interspinous ligaments, the TLF and in the paravertebral lumbar musculature. Patients were evaluated after six months, and in those who received one intervention, an average of 36% improvement in pain intensity (VAS) was reported, but with no concomitant functional improvement, while in those receiving two interventions, the reduction in the level of pain was 48%, together with a 30% improvement in the functional index used.

As opposed to this study, we used a strict protocol with two interventions at fixed time points, using US guidance to confirm correct intralesional placement. Moreover, we chose a US confirmed soft tissue pathology as an inclusion criterion. Compared to Darrow's results we report a 70% reduction in pain as well as improvement in spinal mobility. This greater alleviation of pain might be a result of our stricter inclusion criteria. While Darrow et al. included patients with chronic, “non-specific” lumbar pain—a broad term, our study population consisted exclusively of patients diagnosed with ICPS as a cause for their LBP. This fact should further stress the importance of recognizing specific pain generators when treating patients with LBP, avoiding the use of the umbrella term “non-specific LBP”.

Hussein et al. (45) investigated the effect of PRP injections in LBP accompanied by evidence of atrophy or hypotrophy of the paraspinal muscles in mono-segmental degenerated intervertebral disc disease. Patients were injected PRP monthly for 6 months and were followed for a period of 24 months thereafter. The Pain Intensity Index (NRS) in patients gradually significantly improved from 8.8 to 3.5. The same was true for the improvement in the functional capacity (Oswestry Disability Index)—from 36.7 to 14.6 on the twelfth month. This study is important, as it assessed simultaneously both domains of LBP, i.e., pain and function.

In another pilot study evaluating the efficacy of PRP in soft tissue pathology causing LBP, Cameron et al. (46) injected PRP subfascialally (inferior to the TLF) in several points—the sacral region, the paravertebral muscles and axial in the lumbosacral spine. Authors reported significant improvement of VAS—by 77%, with 68% of patients having more than 75% improvement in their symptoms.

These previous studies, though heterogenous, generally lead to two important conclusions. The first one is that soft tissue pathology is truly an important and frequent cause of chronic low back pain and the second one—that injecting PRP could represent a valid treatment in this situation. The main difference between them and our study lays in the fact that we selected a homogeneous group of LBP patients—with clinical features of ICPS and ultrasound confirmed soft tissue pathology around the PSIS, as well as we used a strict injection protocol—two injections three weeks apart at the same location under ultrasound guidance. These more rigid criteria allowed for better analysis of the PRP therapy. From our results we can conclude that the PRP is an efficient treatment for LBP originating from ICPS. In addition, there were no significant adverse reactions or side effects recorded through the study. This therapy was very well accepted by the patients and only a minor periprocedural pain on both interventions were reported by the participants.

However, our study has several limitations. Firstly, there was an obvious female patients' predominance, as more women were diagnosed with ICPS. This was noted also in other studies of ICPS and a similar disorder—the great trochanteric pain syndrome and the posterior pelvic pain syndrome, the female predominance was marked and pointed out by all the researchers (10, 47). Possible explanations include the anatomical features of the female pelvis, with increased insertional angles of the ES, ESA and TLF and glutei tendons, as well as the mode of walking and the widening of the pelvis with age. It was noted that the bony pelvis widens over 20 mm between the ages of 20 and 80, more in women, and while the pelvic inlet does not enlarge over time, the distance between the hips significantly increases (48). Another limitation could be the relatively short follow-up. Still, the six-month period is relevant in the clinical practice, as during longer periods, other injuries are likely to occur, confounding the results and the effect of the specific treatment. Thirdly, we could not perform a platelets cell count in the ready PRP after concentration because of logistic issues and instead relied on the manufacturer product specifications. As the manufacturer had a valid FDA approval for several years at the time of the study, we decided that we can use its data in our cell count calculation on the approximate number of platelets delivered intralesional during the therapy. Another limitation of our study is the relatively small sample size and single arm design that could limit the generalization of our findings. This was influenced by two factors beyond our control. The amount of the postdoctoral research grant that funded the study and the early post Covid time, when the study started, made some patients reluctant to participate. The results, however, are encouraging and could lead to a larger scale randomized trial on the treatment of ICPS. Lastly, we have neither MRI nor histological confirmation of the tendo-ligamentous changes in our patients with ICPS. However, MRI has certain limitations in this pathology as degenerative entheses and ligaments are relatively avascular structures. Besides, our previous comparative study between MSUS and MRI in ICPS showed that MSUS is superior in identifying this condition (49). Surgery is currently not a valid treatment for ICPS; thus, histology samples could not be obtained for ethical reasons.

## Conclusions

Our results show that US guided intralesional injections of PRP in the soft tissue structures around the PSIS, namely the ES, ESA, TLF and LPSL entheses, could potentially be an efficacious and safe treatment option in ICPS. However, future well designed randomized control trials with larger sample size and longer follow up are needed to really assess efficacy and long-term effectiveness of PRP in the treatment of ICPS and associated LBP.

## Data Availability

The original contributions presented in the study are included in the article/supplementary material, further inquiries can be directed to the corresponding author/s.

## Supplementary material

The Supplementary Material for this article can be found online at: https://www.frontiersin.org/articles/10.3389/fmed.2026.1943518/full#supplementary-material

Supplementary Fig 10A Sagittal and slightly oblique (left to right) MRI of the right PSIS. Abbreviations ES - Erector spinae muscle; ES-Erector spine enthesis; CF – Caput femuris; GM – Gluteus maximus; sac - sacrum; *** -Long Posterior Sacroiliac Ligament; open arrow – Thoracolumbar Fascia enthesis.

Supplementary Fig 10B Sagittal and slightly oblique (left to right) MRI of the left PSIS. Abbreviations ES - Erector spinae muscle; ES-Erector spine enthesis; GM – Gluteus maximus; sac - sacrum; sij – sacroiliac joint; open arrow – Thoracolumbar Fascia enthesis.

Video 1 Procedure step 1 - PRP intralesional injection targeted to the ES/TLF entheses.

Video 2 Procedure step 1 - US guided intralesional PRP injection targeted to the ES/TLF entheses.

Video 3 Procedure step 2 - PRP intralesional injection targeted to the LPSL.

Video 4 Procedure step 2 - Ultrasound image of a PRP intralesional injection targeted to the LPSL.
